# Exon 1 deletion of the androgen receptor gene causing complete androgen insensitivity syndrome in a newborn: a case report

**DOI:** 10.3389/fped.2025.1508618

**Published:** 2025-04-25

**Authors:** Shengxia Wang, Ya-Ting Zhang, Fan Wang

**Affiliations:** Department of Neonatology, The Second Hospital of Lanzhou University, Lanzhou City, Gansu Province, China

**Keywords:** CAIS, 46, XY, new mutation site, case report

## Abstract

**Objective:**

To genetically characterize a case of neonatal complete androgen insensitivity syndrome (CAIS) and identify the underlying molecular defect.

**Methods:**

This was a retrospective analysis of the clinical data, diagnosis, and treatment of a case of CAIS in the Second Hospital of Lanzhou University. Genetic testing of the patient and their parents was done; the pathogenic genes of the child were detected using whole exome sequencing (WES) technology.

**Results:**

The social sex of the proband was female, but the chromosomal sex was male. WES detected Exon 1 deletion mutation of AR gene in the proband and Exon 1 heterozygosity deletion in the mother. This mutation may cause disease according to the ACMG guidelines, but this variation has not been reported in CAIS caused by an AR gene.

**Conclusion:**

This study genetically characterized a neonate with CAIS, identifying a novel Exon 1 deletion in the AR gene as the underlying cause. This finding expands the spectrum of known mutations associated with CAIS and provides valuable insights into the genetic basis of this condition.

## Introduction

Androgen Insensitivity Syndrome (AIS,OMIM: 300068) is an X-linked recessive genetic disorder associated with mutations in androgen receptor (AR) coding genes. It is a rare sexual development disorder with an incidence of 2/100,000–5/100,000 (1). According to its clinical severity, AIS is divided into complete androgen insensitivity syndrome (CAIS) with typical female external genitalia, partial androgen insensitivity syndrome(PAIS) with dominant male external genitalia or ambiguous external genitalia, and mild androgen insensitivity syndrome(MAIS) with typical male external genitalia or an isolated small penis. In adolescence, it manifests as gynecomastia, while it presents as infertility in adulthood (2). Among the different types, CAIS is the most common and most serious type, with an incidence of 1 in 20,400–99,100 (3); it is characterized by a male karyotype (46,XY), cryptorchidism, female external genitalia, and secondary sexual characteristics (4, 5). There are more than 1,000 different AR gene variants that cause the disease. Due to various clinical manifestations, it is easy to misdiagnose or miss the diagnosis entirely. This report retrospectively analyzes a case of CAIS admitted to the Department of Neonatology of the Second Hospital of Lanzhou University, in the hopes of clarifying the etiology, diagnosis, and treatment of this rare disease.

## Clinical data

The patient is a 4-day-old female admitted to our hospital for 4 days due to a 4-day history of postnatal vomiting and 3-day history of labored breathing with sputum expectoration. Initially, the patient presented with vomiting, labored breathing, sputum expectoration, and cyanosis during postnatal feeding. The patient was managed with invasive ventilator assisted ventilation while seeking treatment at another hospital. Incidentally, the insertion of a 10 cm gastric tube was blocked, and complete gastrointestinal angiography indicated congenital esophageal atresia with tracheoesophageal fistula. The patient was transferred to the neonatology department of our hospital as a case of esophageal atresia with esophageal tracheoesophageal fistula. The patient was born full-term to a G3P3 mother via spontaneous vaginal delivery with a birth weight of 3.6 kg. She has two healthy sisters, aged 10 and 6 years, neither of whom has reached menarche. The parents were nonconsanguineous, healthy, and denied any family history of congenital developmental malformations or other diseases The patient had a length of 55 cm, head circumference of 37 cm, chest circumference of 35 cm, and body weight of 3.31 kg. Physical examination revealed yellow skin, auditory and moist rales in both lungs, audible and grade II/6 murmurs in the precardiac area, no abnormalities in the abdomen, anus and external genitalia. The patient was positive for the primitive reflex, but negative for the pathological reflex.

**Figure 1:**
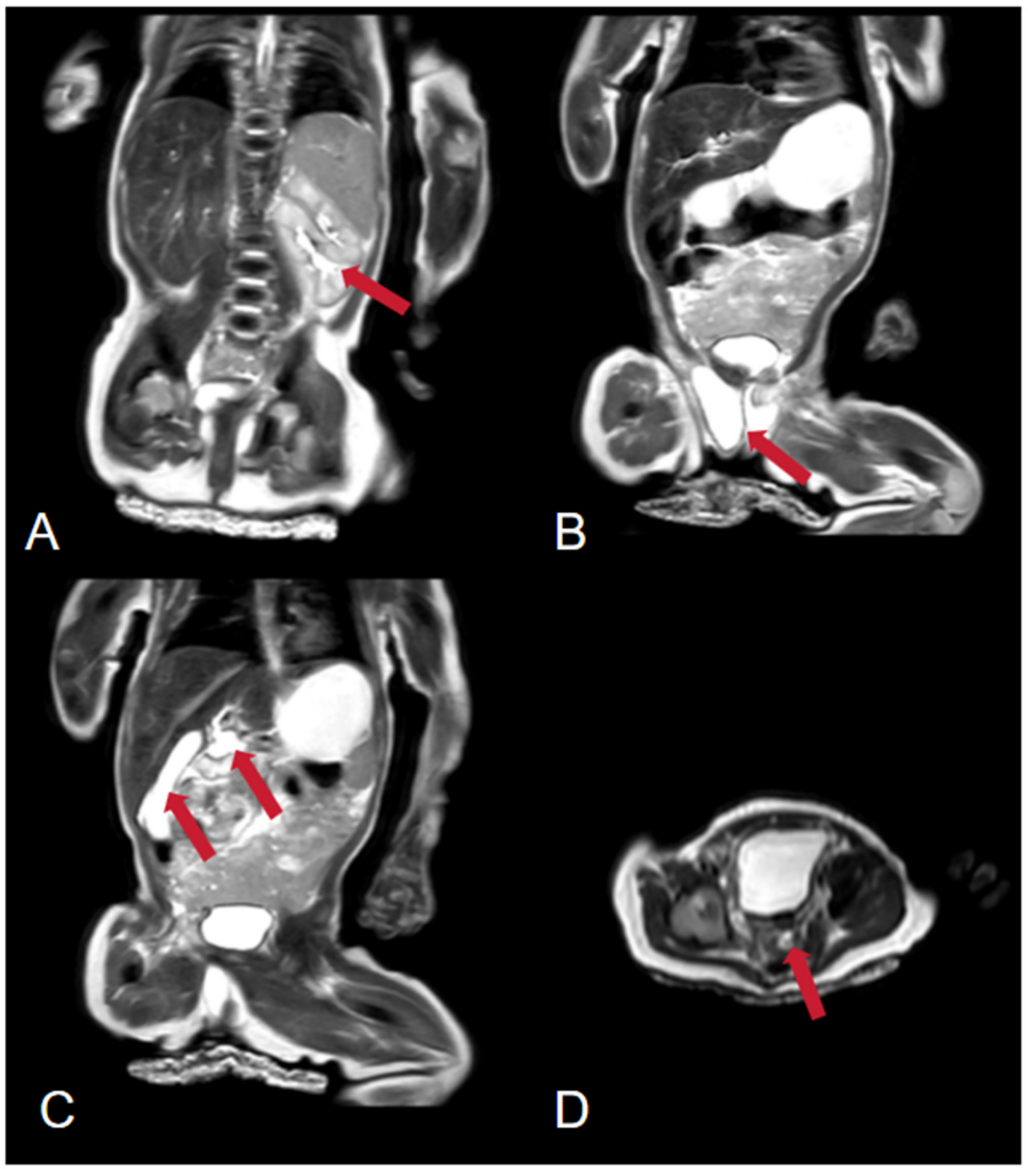
Abdominal magnetic resonance imaging (MRI). **(A)** The arrow points to a duplicated left kidney. **(B)** The arrow points to the testes. **(C)** The arrow points to the gallbladder and bile duct tissue. **(D)** The arrow points to a suspected uterine signal.

**Figure 2:**
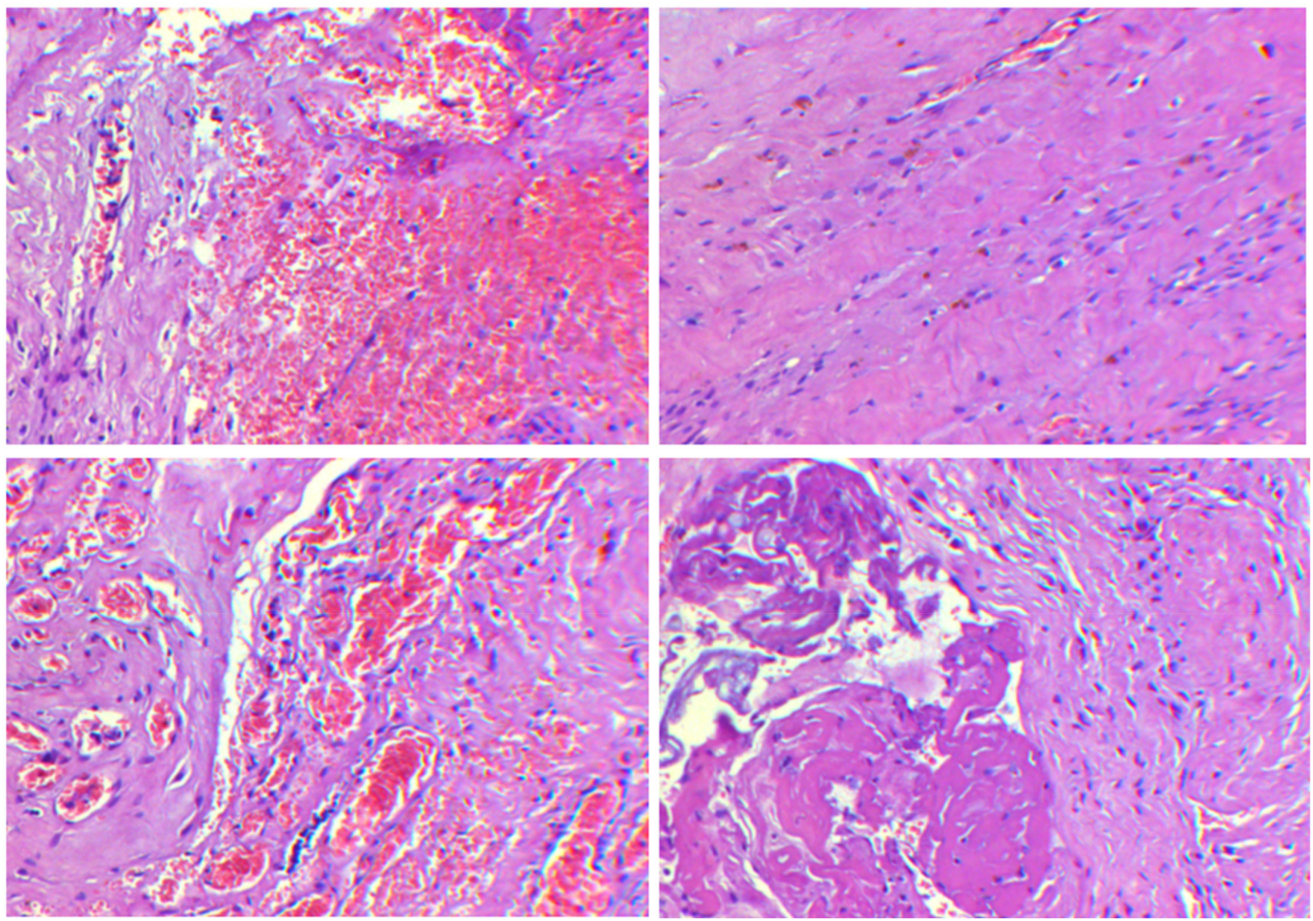
Testicular biopsy results at different fields of view show the presence of fibrous hyperplasia, accompanied by small vessel proliferation, dilation, and congestion, with focal hemorrhage as well as signs of degeneration and necrosis.

The pertinent laboratory findings upon admission were as follows: total bilirubin (TBIL) of 276.8 umol/L, direct bilirubin (DBIL) of 26.9 umol/L, indirect bilirubin (IBIL) of 249.9 umoI/L, alanine aminotransferase (ALT) of 8 U/L, and aspartate aminotransferase (AST) of 23 U/L. Color Doppler ultrasound indicated a patent ductus arteriosus. Pulmonary ultrasound revealed fragmentary consolidation of both lungs, left pleural effusion, and diffuse fusion of both lungs on line B. After assisted ventilation with an invasive ventilator and intravenous nutrition, serum bilirubin levels decreased, and thoracoscopic esophagostomy was performed. Postoperatively, anti-infection measures were taken, and intravenous nutrition was provided. Bilirubin and aminotransferase levels were monitored, with the following results: TBIL: 324.5 umol/L, DBIL 258.6 umol/L, IBIL: 65.9 umoI/L, ALT: 343 U/L, AST: 654 U/L. Upon further investigating the cause of the increased bilirubin, an abdominal mass was palpated. Magnetic resonance imaging (MRI) indicated the absence of the right kidney, a repeated kidney on the left side, a large gallbladder volume, and dilatation of common bile duct (Figure 1). Accompanying these congenital changes were a poor descent of both testes and hydrocele, as well as uterine signal shadows on the right side and in the pelvic cavity. Due to the suspected Differences of Sex Development (DSD), endoscopic exploration, cholecystostomy, biliary irrigation, and right testicular tissue biopsy were performed. Postoperative reexamination revealed that bilirubin and transaminase levels did not decrease significantly (TBIL: 385.1 umol/L, DBIL: 270.8 umol/L, IBIL: 114.3 umoI/L, ALT: 179 U/L, AST: 246 U/L). Pathological analysis (Figure 2) revealed that the tissue submitted for examination was immature testicular tissue, and this disease could not be ruled out due to genetics. The parents claimed that there was no similar disease in the family. After approval by the Medical Ethics Committee of the Second Hospital of Lanzhou University (Project Number: 2024A-929) and informed consent of the parents, genetic testing of the child was performed to find the disease-causing mutation site.

## Methods and analysis

**Figure 3:**
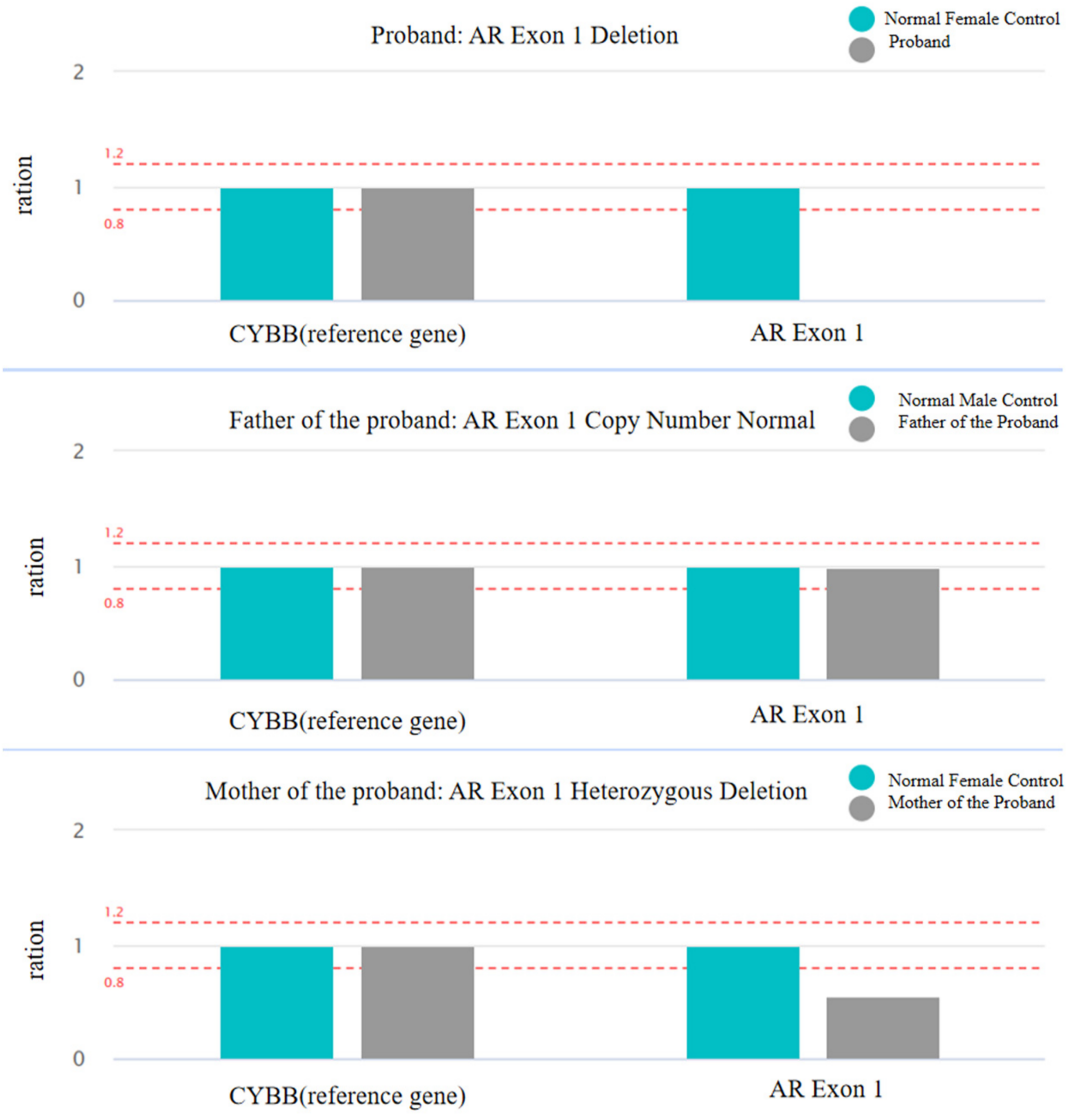
Copy number variation (CNV) of AR Exon 1 deletion was assessed using qPCR. The figure illustrates the relative copy number ratios of AR Exon 1 and the CYBB reference gene in the proband and their parents, evaluating the copy number variation of AR Exon 1.

Blood samples of the patient and her parents were extracted and sequenced via whole Exome sequencing using the IDT xGen Exome Research Panel v2.0 full exon chip capture high-throughput sequencing technology. This yielded an original data output of 16,790 Mb, probe coverage length of 42.5 Mbp, target coverage of 99.80%, average depth of >20 X, and coverage of 99.10%. The analysis and screening of genetic disease was performed using the precision diagnosis cloud platform system, pathogenic mutation database, normal human genome database, clinical characteristics database of 4,000 known genetic diseases, gene data analysis algorithm, three-factor classification system, and ACMG gene variation classification system. After PCR amplification of the target sequence, candidate mutation sites were validated through next-generation sequencing (NGS), and the results were obtained via sequence analysis software. Whole exome sequencing (WES) revealed a deletion mutation in AR gene Exon 1 (Table 1, reference genome: hg19). The patient was a 46,XY male with normal chromosome count, suggesting that the AR gene mutation is associated with the patient's phenotype. Quantitative PCR (qPCR) was used to detect copy number variation (CNV) in AR Exon 1, and the results indicated that the Exon 1 deletion mutation was inherited from the mother (Figure 3). According to the ACMG guidelines, this mutation may cause disease and is not included in the normal population database or gnomAD database, making it a rare mutation.

**Table 1:** Whole exome sequencing results (reference genome: hg19).

Item	Results
Chromosomal location	chrX:66764988-66766604
Nucleotide alteration (exon)	loss (Exon:1)
Amino acid variant (transcript)	-(NM 000044)
Pathogenic classification	LP
Homozygous proband (Male)	Deletion
Homozygous father (Normal)	wild-type
Homozygous mother (Normal)	Heterozygous deletion
Mode of inheritance	1. Androgen Insensitivity Syndrome (AIS) (OMIM: 300068), X-linked Recessive (XR) 2. Partial Androgen Insensitivity Syndrome (PAIS) (OMIM: 312300), X-linked Recessive (XR) 3. X-linked Hypospadias Type 1(HYSP1) (OMIM: 300633), X-linked Recessive (XR) 4. Kennedy's Disease/X-linked Spinal and Bulbar Muscular Atrophy Type 1 (SMAX1) (OMIM: 313200), X-linked Recessive (XR)

## Discussion

AIS, also known as testicular feminization syndrome, was proposed in 1953 by Morris J M et al. (6); it is an X-linked recessive genetic disease caused by mutations in the AR gene located in the Xq11-q12 region (7). The AR gene is a single chain polypeptide composed of four main functional domains: the (1) N-terminal domain (NTD), (2) DNA binding domain (DBD), (3) hinge domain, and (4) C-terminal ligand-binding domain (LBD) (8–12). CAIS is the inactivation mutation of the AR gene, resulting in the absolute insensitivity of embryonic tissue to androgens, differentiation of Wolff tube and urogenital sinus in such a way that causes male genital duct obstruction, and the restriction of testicular descent into cryptorchidism, which can be located in any part of the descending testicular pathway (e.g., abdominal pelvis, inguinal canal, and labia major) (13). Additionally, the serum estradiol level of AIS patients under the action of high gonadotropin is much higher than that of normal males, and it is enough to induce female secondary sexual characteristics and maintain female body shape characteristics. Thus, patients have male internal genitalia and female external genitalia (14). Most patients with CAIS are usually diagnosed during adolescence when they present for primary amenorrhea or inguinal hernia, while diagnosis in the neonatal period is rarer.

With the widespread use of modern molecular biology techniques and genetic testing, over 1,000 different mutations in the AR gene have now been reported (15), with new mutation sites continuously emerging. In a study by Susanne Ledig et al. (16), 12 novel mutations were identified in 24 AIS patients, most of which were located in exons encoding the DNA-binding domain and ligand-binding domain. Additionally, it has been established that the primary cause of AIS is missense mutations in the AR gene, particularly within Exon 1. Mutations in Exon 1 are frequently associated with the formation of premature stop codons in cases of CAIS (17). The deletion of Exon 1 leads to the loss of the N-terminal domain (which includes the transcriptional activation function 1, or AF-1, region), resulting in impaired androgen receptor functionality. This loss disrupts the receptor's ability to bind DNA and activate the transcription of target genes, both of which are essential for normal androgen signaling and physiological development.In addition, alterations in the CAG repeat sequence in exon 1 of the AR gene are closely related to AR transcriptional activity (18). Also for example, epigenetic modification sites, such as m6A RNAs undergoing modification affect AR mRNA translation (19).

The patient was a phenotypic female with no family history of CAIS who presented with esophageal atresia with esophagotracheal fistula. Since DSD is often overlooked due to the presence of normal female external genitalia, cases of CAIS can be missed during the initial diagnosis and treatment of esophageal atresia. Due to the abnormal bilirubin index, a more thorough physical examination revealed a palpable abdominal mass, and further imaging and pathological biopsy indicated the presence of testicular tissue. Chromosomal analysis of the peripheral blood revealed a 46,XY genotype, and WES revealed an AR gene deletion (Exon 1 deletion). CAIS was eventually diagnosed as being caused by an Exon 1 deletion mutation.

In clinical practice, more common diseases are considered first. Thus, a neonate with normal external genitalia and an abdominal mass, will not automatically raise suspicion for AIS. The diagnosis of AIS can be missed during routine laboratory examinations, since making the diagnosis often requires an in-depth work-up. This paper summarizes the diagnosis and treatment of a neonate initially diagnosed with esophageal atresia with esophagotracheal fistula and abnormal bilirubin indexes for unknown reasons, until finally being diagnosed with CAIS. This report aims to increase the awareness of CAIS, especially regarding its diagnosis and treatment. Our key findings are as follows. (1) In this case, the child had DSD, bilirubin elevation of unknown cause, congenital esophageal atresia, and the chromosome test showed 46,XY, and the diagnosis of CAIS was finally made by WES; however, the results of the peripheral blood genetic test in this case could not confirm the characteristics of the multisystemic malformations that the child showed. Whole-exome sequencing mainly focuses on the exon region, with limited ability to detect variants in non-coding regions, making it easy to miss the detection of important functional loci. Abnormalities in epigenetic modifications may also be present, so methylation testing may also be performed in anticipation of improved diagnostic accuracy. (2) Although karyotype analysis did not reveal any chromosomal abnormalities accounting for the phenotype, given the X-chromosomal localization of the AR gene and its pivotal role in sexual development, high-resolution chromosomal microarray analysis (CMA) is recommended to systematically screen this region for potential rearrangements, microdeletions, or copy number variations to refine the molecular diagnosis. (3) Although CAIS patients typically exhibit testes capable of secreting AMH and testosterone, the histopathological findings in this case revealed immature testicular tissue, a feature inconsistent with classic CAIS. We hypothesize that this developmental arrest may result from dysfunctional AR signaling due to the identified mutation, impairing testicular maturation. This aligns with the known role of AR in testicular development, where loss-of-function mutations disrupt Wolffian duct stabilization, leading to absent or hypoplastic structures such as the epididymis and vas deferens, as documented in prior studies (20). (4) For newly admitted children, a careful physical examination is essential. If an abdominal mass is found, aside from hernia, undescended testes should be considered. Relevant investigations should be further refined to clarify the nature of unknown masses. (5) Although the patient underwent multiple operations during treatment, the bilirubin levels remained elevated. No gene mutation that may lead to abnormalities was found after further genetic examination, and the reason is still unclear. Due to financial reasons, the family decided to give up treatment, and the patient was discharged automatically without identifying the cause. However, upon followup 1 month after discharge, the patient had normal physical development, could tolerate mixed feeding, and had significantly decreased bilirubin levels. Therefore, the abnormal bilirubin in this child may be attributed to liver injury caused by parenteral nutrition support during hospitalization. Therefore, to reduce liver injury, physicians should consider early reintroduction of enteral nutrition to avoid overfeeding, supplementation with cholelitic drugs, and appropriate use of antibiotics or probiotics.

In summary, the child in this case was diagnosed with Complete Androgen Insensitivity Syndrome (CAIS) caused by an Exon 1 deletion mutation in the AR gene. The deletion of Exon 1 leads to a complete loss of AR gene function, presenting with the typical clinical phenotype of CAIS. Although no features related to multiple congenital abnormalities associated with esophageal atresia and bilirubin abnormalities were found, the clinical presentation of the child is consistent with the diagnosis of CAIS. Typically, children with CAIS are raised as females. It is important to note that ectopic testes, particularly those located in the abdominal cavity, may have an increased risk of malignancy due to long-term exposure to relatively high body temperature, although the risk of testicular cancer before puberty is low. Furthermore, testosterone and estrogen secreted by the testes play an important role in adolescent height growth and the development of female secondary sexual characteristics. Therefore, orchiectomy is recommended after the development of secondary sexual characteristics. Long-term follow-up and support for the child's mental health and social adaptation are also necessary, which will provide valuable reference for future diagnosis and treatment.

## Data Availability

The datasets presented in this study can be found in online repositories. The names of the repository/repositories and accession number(s) can be found in the article/Supplementary Material.
